# Prognostic potential of standard laboratory parameters in patients with metastatic renal cell cancer receiving first-line immunotherapy

**DOI:** 10.1038/s41598-024-76928-3

**Published:** 2024-10-25

**Authors:** Bjoern Thorben Buerk, Cathrin Kusiek, Vayda Schüttke, Marcus Sondermann, Abdulbaki Yakac, Elena Abbate, Susanne Fuessel, Christian Thomas, Kati Erdmann

**Affiliations:** 1 Department of Urology, Faculty of Medicine, University Hospital Carl Gustav Carus, Technische Universität Dresden, Dresden, Germany; 2 German Cancer Consortium (DKTK), Partner Site Dresden, Dresden and German Cancer Research Center (DKFZ), Heidelberg, Germany

**Keywords:** Early kinetics, γ-Glutamyltransferase, Immune checkpoint inhibitors, Lactate dehydrogenase, Prognostic biomarkers, Metastatic renal cell cancer, Prognostic markers, Renal cell carcinoma, Translational research, Cancer immunotherapy

## Abstract

Through their involvement in cancer metabolism, alanine aminotransferase (ALAT), aspartate aminotransferase (ASAT), γ-glutamyltransferase (GGT) and lactate dehydrogenase (LDH) reflect the tumor burden and thus could have a prognostic potential for patients treated with immune checkpoint inhibitors (CPI). Therefore, this study investigated the prognostic potential of these parameters in a real-world cohort of patients with metastatic renal cell cancer (mRCC) under first-line CPI-based therapy. The retrospective study cohort included 82 mRCC patients treated with CPI-based first-line therapy between 2019 and 2023. Progression-free survival (PFS), overall survival (OS) and response rates were evaluated according to baseline levels and early dynamic changes of ALAT, ASAT, GGT and LDH. Multivariate Cox proportional hazard regression models were generated to identify independent prognosticators for PFS and OS. High baseline levels and non-normalized kinetics of ALAT, ASAT, GGT and LDH were significantly associated with shorter PFS and OS (p < 0.05), which was also reflected by lower response rates. Combining the four parameters at baseline into a 4-Risk-Score resulted in an enhanced prognostic power, as indicated by a higher C-index of 0.693 for OS compared to the individual parameters (≤ 0.663). Patients with all four risk factors present showed the worst PFS and OS. Overall, baseline levels and early kinetics of the four parameters as well as the 4-Risk-Score were identified as independent prognosticators for PFS and OS by multivariate analysis. As standard laboratory parameters, ALAT, ASAT, GGT and LDH are cost-effective and could be easily used either alone or in combination for therapy monitoring of CPI-treated mRCC patients.

**Supplementary Information:**

The online version contains supplementary material available at 10.1038/s41598-024-76928-3.

## Introduction

Renal cell carcinoma (RCC) is the 3rd most frequent urological cancer with a high risk of metastasis^1,2^. About 30% of the patients are diagnosed with metastatic disease (mRCC), and another 30–40% will develop metastases after initially being treated with curative intent^1^. Immune checkpoint inhibitors (CPI) have revolutionized the treatment of mRCC by augmenting the endogenous anti-tumor response leading to improved survival compared to standard anti-angiogenic therapy with tyrosine kinase inhibitors (TKI)^3,4^. CPI-based first-line (1L) therapy of mRCC patients currently includes only combinations with either TKI or other CPI, whereas CPI monotherapy with nivolumab is used in subsequent treatment lines (≥ 2L) after TKI monotherapy^3,5^. Recently, the CPI pembrolizumab has also been approved in the adjuvant therapy setting of RCC patients at an increased risk of recurrence after surgery^6,7^.

CPI-based therapy can be accompanied by (i) severe immune-related adverse events (irAEs) due to an excessively augmented activity of the immune system and (ii) highly variable therapy responses due to primary or secondary resistance, both of which are currently unpredictable^8,9^. Therefore, reliable biomarkers are urgently needed to better stratify patients for CPI therapy. Standard laboratory parameters might act as prognostic and/or predictive biomarkers in this context. They are already implemented into the clinical routine and thus could easily facilitate therapy monitoring. In accordance, we have previously demonstrated that C-reactive protein (CRP), particularly its dynamic change in the early therapy phase, has a high prognostic potential for mRCC patients on CPI-based 1L therapy^10,11^.

Alanine aminotransferase (ALAT), aspartate aminotransferase (ASAT) and γ-glutamyltransferase (GGT) are predominantly used as diagnostic markers for liver dysfunction, while lactate dehydrogenase (LDH) indicates tissue damage. Furthermore, all four parameters are strongly involved in cancer metabolism and can promote tumor progression, an immunosuppressive microenvironment and/or drug resistance^12–17^. Hence, various studies have also reported a prognostic value for the ASAT/ALAT ratio (= De Ritis Ratio; DRR), GGT and LDH in surgically treated RCC patients^18–22^. To date, the prognostic role for CPI-based therapy in mRCC has only been investigated for GGT and LDH and solely in patients treated by nivolumab in ≥ 2L, where high serum levels were a risk factor for poor prognosis^23–26^. To the best of our knowledge, ALAT, ASAT or DRR have not been evaluated as prognosticators in mRCC patients on CPI-based therapy. Therefore, this study aimed to evaluate the associations of the baseline levels and early changes of ALAT, ASAT, DRR, LDH and GGT with survival and response in a real-world cohort of mRCC patients on CPI-based 1L therapy.

## Methods

### Patients

A total of 82 mRCC patients were initiated on CPI-based 1L therapy between March 2019 and December 2023 at the Department of Urology (University Hospital Dresden). The treatment was selected according to the effective guidelines at the time^27^and conducted until either disease progression or intolerable adverse events were observed. Computed tomographic or magnetic resonance imaging of the chest, abdomen and pelvis were performed every three months in accordance with the current German RCC guideline^27^. Additional imaging was done depending on the patient’s condition. The best overall response (BOR) to treatment was determined according to the response evaluation criteria in solid tumors (RECIST) version 1.1 and categorized as complete response (CR), partial response (PR), stable disease (SD) or progressive disease (PD)^28^. Response to treatment was defined as CR and PR. The respective clinical and laboratory data were retrospectively retrieved from the patients’ medical records. The censor date for the patients’ follow-up was 31 May 2024. Data collection and analysis were approved by the institutional review board of the Technische Universität Dresden (BO-EK-107032023) and conducted according to the Declaration of Helsinki. Written informed consent was obtained from the patients.

### Assessment of laboratory parameters and definition of early kinetics

Serum levels for ALAT, ASAT, GGT and LDH were determined in an accredited routine laboratory at baseline (< 4 weeks prior to treatment start) and thereafter regularly at patient visits. DRR was calculated as the ASAT/ALAT ratio. Values from hemolyzed serum samples were excluded from further analysis, since hemolysis can influence the accuracy and reliability of routine chemistry testing. Patients were then stratified based on the respective median baseline level into high (> median) and low (≤ median). Subsequently, ALAT, ASAT, GGT and LDH were combined into the 4-Risk-Score according to the respective baseline stratification, which resulted in possible risk scores of 0 to 4. For early change of all parameters within the initial three months of 1L therapy, patients were classified as normal (low baseline), normalized (high baseline and low nadir within three months) and non-normalized (high baseline and high nadir within three months). This kinetics classification was done in the style of CRP kinetics previously published^10,11^. For patients with a 1L duration < 3 months, the respective nadir was determined during their entire 1L treatment duration.

### Statistical analysis

Statistical analyses were carried out using IBM SPSS Statistics 29.0.0.0 (IBM, Armonk, NY, USA) and GraphPad Prism 10.2.2 (GraphPad Software, San Diego, CA, USA). The Mann-Whitney-U test (two groups) or the Kruskal-Wallis test (three groups) were used for inter-group comparisons of continuous variables. Relations between two laboratory parameters were evaluated by determination of Pearson correlation coefficients (r). Progression-free survival (PFS) was defined as the time from treatment start to disease progression or death from any cause, whereas patients without disease progression or death were censored at last contact. Overall survival (OS) was defined as the period from treatment start until death from any cause, and patients lost to follow-up were censored at the time of last confirmed survival. The Kaplan-Meier method and the log-rank test were used to evaluate differences between groups regarding PFS and OS. Survival rates for PFS and OS after one and two years were determined by life tables. Via univariate and multivariate Cox regression analyses, hazard ratios (HR) including their 95% confidence intervals (95% CI) and Harrell’s concordance index (C-index) were calculated in order to identify prognostic factors for PFS and OS. Multivariate Cox regression analysis for each laboratory parameter was performed by co-adjusting for the known mRCC prognostic factors Eastern Cooperative Oncology Group performance status (ECOG), International mRCC Database Consortium (IMDC) risk, prior nephrectomy and liver metastasis^29,30^. A p value < 0.05 indicated statistical significance, whereas p values ≥ 0.05 and < 0.1 were considered statistical trends.

## Results

### Patient cohort

Overall, 82 patients on CPI-based 1L therapy were included in this study and their characteristics are summarized in Table 1. The majority of the patients (*n* = 66, 80%) received a CPI + TKI combination, while 16 patients (20%) were initiated on CPI + CPI therapy. The median patient age was 67 years. Most patients were male (72.0%) and had an ECOG of 0–1 (75.6%) and an intermediate or poor IMDC risk (90.2%). The median time from histological RCC diagnosis to 1L therapy was 4.1 months with pure clear cell RCC being the most frequent histological subtype (76.8%). At 1L start, most patients (73.2%) presented metastasis in multiple organs and only 11 patients (13.4%) had liver metastasis. At a median of 25.6 months prior to 1L therapy, most patients (62.2%) had a partial or radical nephrectomy. In addition, seven patients (8.5%) received a cytoreductive nephrectomy at a median of 7.0 months (range 4.6–19.5 months) after 1L start due to good therapy response.

**Table 1:** Patients‘ characteristics at start of CPI-based 1L therapy.

Parameter	Category	Total cohort (*n* = 82)
1L therapy (n)	Avelumab + axitinib Nivolumab + cabozantinib Nivolumab + ipilimumab Pembrolizumab + axitinib Pembrolizumab + lenvatinib	16 5 16 21 24
Age (years)	Median (range)	67 (40–82)
Sex (n)	Male Female	59 23
ECOG (n)	0 1 ≥ 2	25 37 20
IMDC risk (n)	Favorable Intermediate Poor	8 43 31
Time from RCC diagnosis to 1L (months)	Median (range)	4.1 (0.2-295.5)
Histology (n)	Clear cell Clear cell mixed Non-clear cell Unknown	63 6 8 5
Metastatic organs at 1L (n)	Single Multiple	22 60
Liver metastasis at 1L (n)	No Yes	71 11
Prior nephrectomy (n)	No Yes	31 51
Time from prior nephrectomy to 1L (months)	Median (range)	25.6 (1.0-295.5)
CPI cycles	Median (range)	11 (2–88)
Follow-up duration (months)	Median (range)	18.8 (1.3–53.6)
BOR (n)	CR PR SD PD Unknown	5 34 25 16 2
Baseline ALAT (µmol/s*l) ^a^	Median (range)	0.32 (0.09–1.48)
Baseline ASAT (µmol/s*l)	Median (range)	0.37 (0.14–2.72)
Baseline DRR ^a^	Median (range)	1.18 (0.44–3.69)
Baseline GGT (µmol/s*l)	Median (range)	0.58 (0.19–9.46)
Baseline LDH (µmol/s*l)	Median (range)	3.78 (1.97–16.49)

^a^Baseline ALAT and thus DRR are missing for one patient.

Data for radiologic treatment response were available for 80 patients. Five (6.1%), 34 (41.5%), and 25 (30.5%) patients achieved CR, PR and SD as BOR, respectively. In contrast, 16 patients (19.5%) did not respond to CPI-based therapy (PD).

The median follow-up, PFS and OS of the total cohort were 18.8, 16.8 and 35.6 months, respectively. Thirty-two patients (39.0%) showed no progression and had ongoing 1L therapy, while one patient on pembrolizumab combined with lenvatinib switched to 2L therapy due to TKI-mediated side effects. Three patients (3.7%) continued 1L therapy after progression and metastasis-directed treatment, 32 patients (39.0%) received 2L therapy after progression, and 14 patients (17.1%) died or progressed without 2L therapy. Overall, 36 patients (43.9%) had died until the censor date (May 2024).

### Baseline levels and early kinetics of laboratory parameters

Baseline levels for ASAT, GGT and LDH were available for all patients, while ALAT and thus DRR were missing for one patient. The median baseline levels were 0.32 µmol/s*l for ALAT, 0.37 µmol/s*l for ASAT, 1.18 for DRR, 0.58 µmol/s*l for GGT and 3.78 µmol/s*l for LDH (Table 1). Subsequently, the respective median baseline levels were used to stratify patients into groups with high and low ALAT, ASAT, DRR, GGT and LDH.

Baseline ALAT and ASAT levels highly correlated (*r* = 0.652, *p* < 0.001), while ALAT/GGT (*r* = 0.453, *p* < 0.001), ASAT/GGT (*r* = 0.359, *p* = 0.001) and ASAT/LDH (*r* = 0.440, *p* < 0.001) showed an intermediate correlation (Supplementary Table S1). Only baseline GGT was significantly elevated in patients with liver metastasis (Supplementary Table S2). However, it has to be noted that only 11 patients presented liver metastasis at 1L start. A pronounced influence of hepatic comorbidities on the baseline levels of the total cohort could be excluded, since only two patients had liver cirrhosis and steatohepatitis, respectively.

Furthermore, patients were categorized as normal, normalized and non-normalized based on the early change of the respective laboratory parameter within the initial three months of 1L therapy (Supplementary Table S3). Of note, only seven patients (8.5%) displayed normalized kinetics for GGT, whereas kinetics groups were distributed more evenly for all other parameters. Patients in the normal group exhibited significantly lower baseline levels for all parameters than patients in the normalized and non-normalized groups. Within the first three months of therapy, the nadirs of all parameters were significantly lower in the normal and normalized groups than in the non-normalized group. Furthermore, the median number of measurements for each laboratory parameter within the first three months after treatment start ranged between 5 and 7. Except for ALAT, no significant differences in number of measurements could be observed between the three groups.

### Association of baseline levels and early kinetics of laboratory parameters with survival

**Fig. 1:**
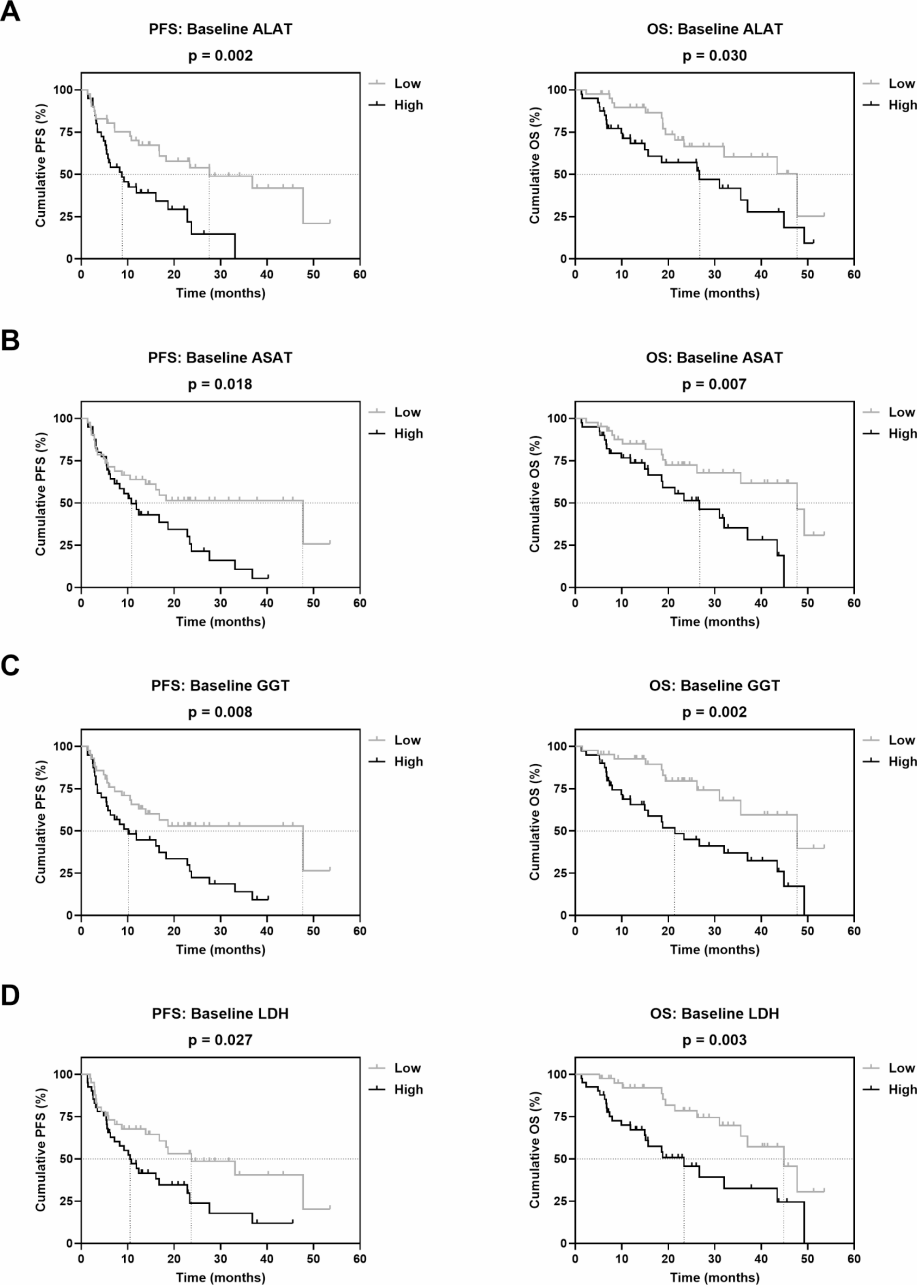
Association of baseline (**A**) ALAT, (**B**) ASAT, (**C**) GGT and (**D**) LDH with PFS and OS of mRCC patients on CPI-based 1L therapy. Vertical dashed lines indicate the respective median survival time of each category. P values were calculated by the log-rank test.

**Fig. 2:**
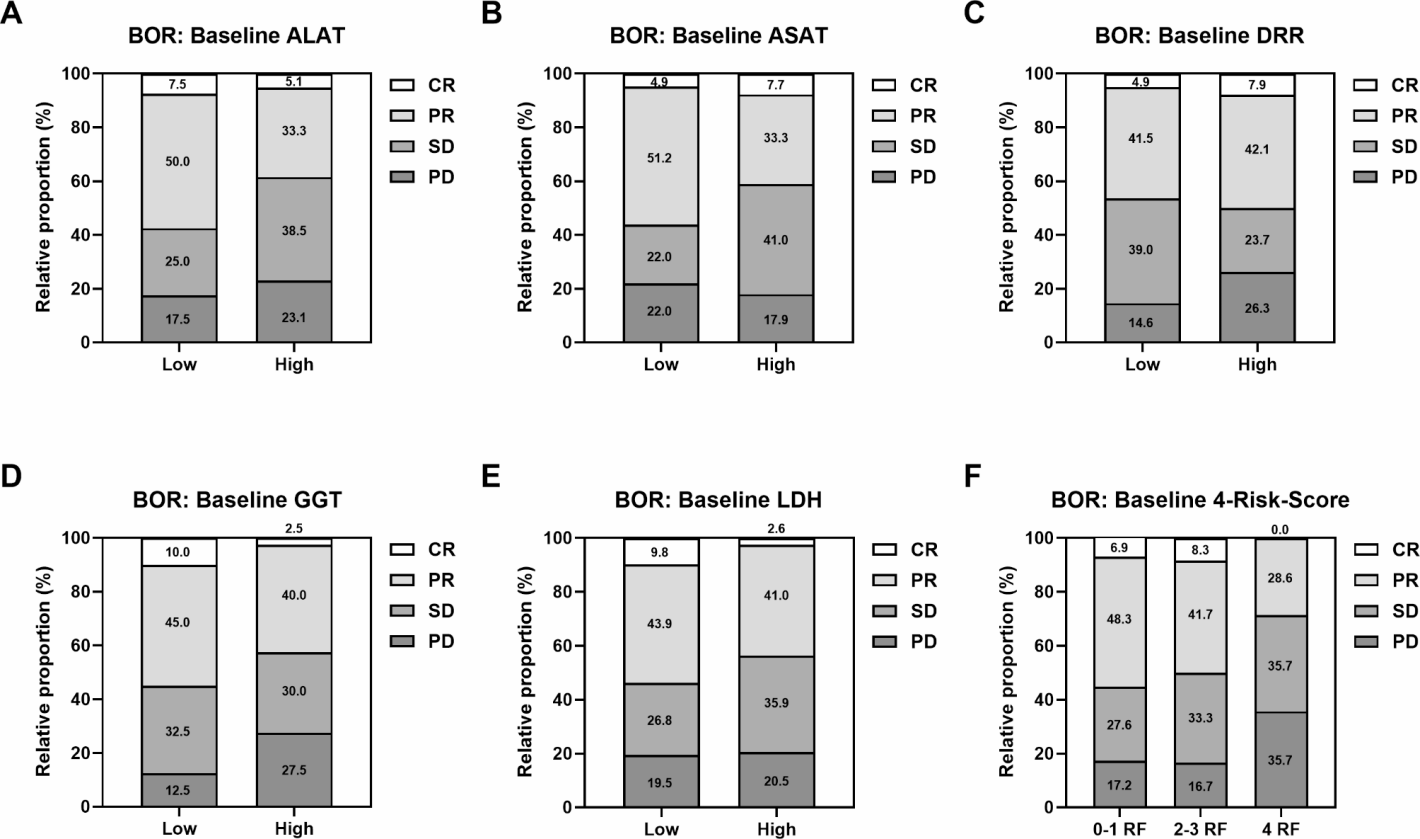
Association of the grouped 4-Risk-Score at baseline (combining ALAT, ASAT, GGT, LDH) with PFS and OS of mRCC patients on CPI-based 1L therapy. Vertical dashed lines indicate the respective median survival time of each category. P values were calculated by the log-rank test.

Patients with low baseline levels of ALAT, ASAT, GGT and LDH showed a significantly longer PFS and OS than patients with high baseline levels (Fig. 1 and Supplementary Table S4). For DRR, only a trend towards a longer OS with low baseline values could be observed (Supplementary Fig. S1A and Supplementary Table S4). Based on the respective baseline stratification of ALAT, ASAT, GGT and LDH, a 4-Risk-Score with 0 to 4 possible risk factors was generated, which was significantly associated with PFS and OS (Supplementary Fig. S2). We then further stratified patients into three prognostic groups according to their risk factors (0–1, 2–3 or 4 risk factors). Patients with four risk factors, i.e., presenting high baseline levels of all four markers, had the worst PFS and OS compared to patients with 0–1 or 2–3 risk factors (Fig. 2 and Supplementary Table S4).

**Fig. 3:**
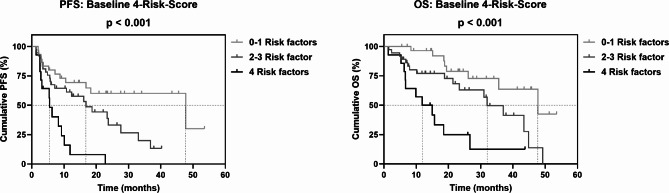
Association of early kinetics of (**A**) ALAT, (**B**) ASAT, (**C**) GGT and (**D**) LDH with PFS and OS of mRCC patients on CPI-based 1L therapy. Vertical dashed lines indicate the respective median survival time of each category. P values were calculated by the log-rank test.

Furthermore, early kinetics of all laboratory parameters, except for DRR, were significantly or per trend associated with survival (Fig. 3 and Supplementary Fig. S1B). In most cases, patients in the normal group had the best PFS and OS, which was associated with higher 1-year and 2-year survival rates (Supplementary Table S4). Except for ALAT, patients with normalized values had a longer median PFS and OS than patients in the non-normalized category (Fig. 3A and Supplementary Table S4).

### Cox regression analysis for baseline levels and early kinetics of laboratory parameters

Results of the survival analysis were confirmed by univariate Cox regression analysis (Supplementary Table S5). Patients with high baseline levels of ALAT, ASAT, GGT and LDH as well as a 4-Risk-Score of 2–3 or 4 had a higher risk for progression and death compared to patients with low values or a 4-Risk-Score of 0–1, respectively. After co-adjusting for known prognostic factors (ECOG, IMDC, prior nephrectomy, liver metastasis) via multivariate Cox regression analysis, baseline ALAT, ASAT, GGT, LDH and the grouped 4-Risk-Score were identified as independent prognostic factors for PFS and OS (Table 2). Furthermore, normal ALAT, ASAT, GGT and LDH kinetics were favorable prognostic factors in univariate Cox regression analyses for PFS and partly for OS (Supplementary Table S5) and emerged as independent prognosticators for PFS and OS in multivariate Cox regression analyses (Table 3).

**Table 2:** Multivariate Cox regression analyses for PFS and OS dependent on baseline levels of ALAT, ASAT, GGT and LDH as well as the grouped 4-Risk-score.

Parameter^a^	Baseline ALAT		Baseline ASAT		Baseline GGT		Baseline LDH		Baseline 4-Risk-Score	
	HR (95% CI)	*P* value	HR (95% CI)	*P* value	HR (95% CI)	*P* value	HR (95% CI)	*P* value	HR (95% CI)	*P* value
**PFS**	C-index: 0.626		C-index: 0.566		C-index: 0.612		C-index: 0.570		C-index: 0.627	
ECOG ≥ 1	0.9 (0.5–1.9)	0.841	0.9 (0.5–1.8)	0.835	1.0 (0.5–1.9)	0.887	1.2 (0.6–2.4)	0.605	1.2 (0.6–2.5)	0.762
Poor IMDC	1.3 (0.6–2.8)	0.470	1.3 (0.6–2.7)	0.546	1.2 (0.6–2.5)	0.659	1.0 (0.5–1.9)	0.874	1.4 (0.7–3.1)	0.524
Prior nephrectomy	1.3 (0.7–2.5)	0.483	1.1 (0.6–2.1)	0.845	1.1 (0.6–2.3)	0.713	1.2 (0.6–2.3)	0.588	1.3 (0.7–2.8)	0.422
Liver metastasis	1.1 (0.4–2.4)	0.819	1.1 (0.4–2.3)	0.918	0.8 (0.3–1.9)	0.632	1.0 (0.4–2.4)	0.926	0.7 (0.2–1.6)	0.516
High baseline level	2.8 (1.5–5.5)	**0.002**	2.1 (1.1–4.1)	**0.019**	2.3 (1.3–4.5)	**0.009**	2.0 (1.1–3.7)	**0.025**		
2–3 Risk factors 4 Risk factors									2.2 (1.1–4.8) 7.2 (2.9–18.1)	**0.037** **< 0.001**
**OS**	C-index: 0.681		C-index: 0.658		C-index: 0.690		C-index: 0.684		C-index: 0.711	
ECOG ≥ 1	2.2 (1.0-5.9)	**0.080**	2.2 (0.9–5.8)	**0.094**	2.2 (0.9–5.8)	**0.097**	2.9 (1.2–7.7)	**0.024**	2.5 (1.0-6.6)	**0.055**
Poor IMDC	1.3 (0.5–3.1)	0.545	1.4 (0.6–3.4)	0.460	1.4 (0.6–3.6)	0.438	1.0 (0.5–2.4)	0.918	1.7 (0.7-4.0)	0.252
Prior nephrectomy	0.7 (0.3–1.6)	0.417	0.7 (0.3–1.6)	0.442	0.8 (0.3–1.7)	0.496	0.8 (0.4–1.8)	0.621	0.9 (0.4-2.0)	0.817
Liver metastasis	2.4 (0.8–6.4)	**0.081**	2.3 (0.8-6.0)	0.110	1.6 (0.5–4.6)	0.368	2.2 (0.7–5.9)	0.147	1.5 (0.5-4.0)	0.490
High baseline level	2.5 (1.2–5.5)	**0.014**	3.0 (1.4–6.8)	**0.005**	3.2 (1.5–7.4)	**0.004**	2.8 (1.4–5.9)	**0.004**		
2–3 Risk factors 4 Risk factors									3.1 (1.3–8.1) 9.3 (3.2–28.1)	**0.016** **< 0.001**

^a^Clinico-pathological parameters (ECOG, IMDC, prior nephrectomy, liver metastasis) and baseline levels of the respective laboratory parameter were included in the multivariate Cox regression model. Reference categories were: ECOG 0, favorable/intermediate IMDC, no prior nephrectomy, no liver metastasis, low baseline levels of individual laboratory parameters, 0–1 risk factors of the grouped 4-Risk-Score.

Significant p values (< 0.05) and statistical trends (*p* ≥ 0.05 & <0.1) are displayed in bold. HR: hazard ratio, CI: confidence interval.

**Table 3:** Multivariate Cox regression analyses for PFS and OS dependent on early kinetics of ALAT, ASAT, GGT and LDH.

Parameter^a^	Baseline ALAT		Baseline ASAT		Baseline GGT		Baseline LDH	
	HR (95% CI)	*P* value	HR (95% CI)	*P* value	HR (95% CI)	*P* value	HR (95% CI)	*P* value
**PFS**	C-index: 0.619		C-index: 0.558		C-index: 0.619		C-index: 0.591	
ECOG ≥ 1	1.0 (0.5–1.9)	0.888	0.9 (0.5–1.8)	0.828	0.9 (0.5–1.8)	0.783	1.2 (0.6–2.4)	0.267
Poor IMDC	1.3 (0.6–2.6)	0.517	1.3 (0.6–2.8)	0.529	1.2 (0.6–2.6)	0.651	0.9 (0.5–1.8)	0.798
Prior nephrectomy	1.4 (0.7–2.8)	0.338	1.1 (0.5–2.1)	0.884	1.1 (0.6–2.2)	0.748	1.2 (0.7–2.4)	0.543
Liver metastasis	0.9 (0.4–2.1)	0.886	1.0 (0.4–2.3)	0.920	0.7 (0.3–1.8)	0.537	0.9 (0.4–2.2)	0.874
Normal kinetics Normalized kinetics	0.5 (0.2–0.9) 1.7 (0.8-4.0)	**0.042** 0.199	0.4 (0.2–0.9) 0.9 (0.4–1.9)	**0.030** 0.740	0.4 (0.2–0.7) 0.6 (0.2–1.4)	**0.004** 0.281	0.4 (0.2–0.8) 0.6 (0.3–1.3)	**0.008** 0.196
**OS**	C-index: 0.692		C-index: 0.660		C-index: 0.696		C-index: 0.684	
ECOG ≥ 1	2.3 (1.0–6.0)	**0.073**	2.2 (0.9–5.8)	**0.098**	2.1 (0.9–5.6)	0.105	2.9 (1.2–7.9)	**0.021**
Poor IMDC	1.3 (0.5-3.0)	0.557	1.4 (0.6–3.6)	0.426	1.4 (0.6–3.5)	0.482	1.0 (0.5–2.3)	0.946
Prior nephrectomy	0.7 (0.4–1.6)	0.441	0.7 (0.3–1.6)	0.414	0.8 (0.3–1.7)	0.490	0.9 (0.4–1.8)	0.677
Liver metastasis	2.3 (0.8–6.1)	0.114	2.3 (0.8–6.1)	0.115	1.6 (0.5–4.5)	0.413	2.0 (0.6–5.6)	0.218
Normal kinetics Normalized kinetics	0.5 (0.2–1.1) 1.3 (0.5–3.3)	**0.084** 0.544	0.3 (0.1–0.7) 0.8 (0.3–1.8)	**0.006** 0.551	0.3 (0.1–0.6) 0.6 (0.2–1.6)	**0.002** 0.351	0.3 (0.1–0.7) 0.7 (0.3–1.8)	**0.006** 0.495

^a^Clinico-pathological parameters (ECOG, IMDC, prior nephrectomy, liver metastasis) and early kinetics of the respective laboratory parameter were included in the multivariate Cox regression model. Reference categories were: ECOG 0, favorable/intermediate IMDC, no prior nephrectomy, no liver metastasis, non-normalized kinetics of laboratory parameters.

Significant p values (< 0.05) and statistical trends (*p* ≥ 0.05 & <0.1) are displayed in bold. HR: hazard ratio, CI: confidence interval.

Of note, the prognostic value of ECOG (C-index 0.551), IMDC risk (C-index 0.602), nephrectomy status (C-index 0.507) and presence of liver metastasis (C-index 0.560) for OS was outperformed by most laboratory parameters, but particularly by GGT and LDH (Supplementary Table S5). For OS, the C-indices for their baseline levels and/or early kinetics ranged from 0.645 to 0.663 (Supplementary Table S5). Moreover, combining the baseline levels of ALAT, ASAT, GGT and LDH into the 4-Risk-Score resulted in a further enhanced prognostic power (C-index for OS 0.693, Supplementary Table S5).

### Association of baseline levels and early kinetics of laboratory parameters with treatment response

**Fig. 4:**
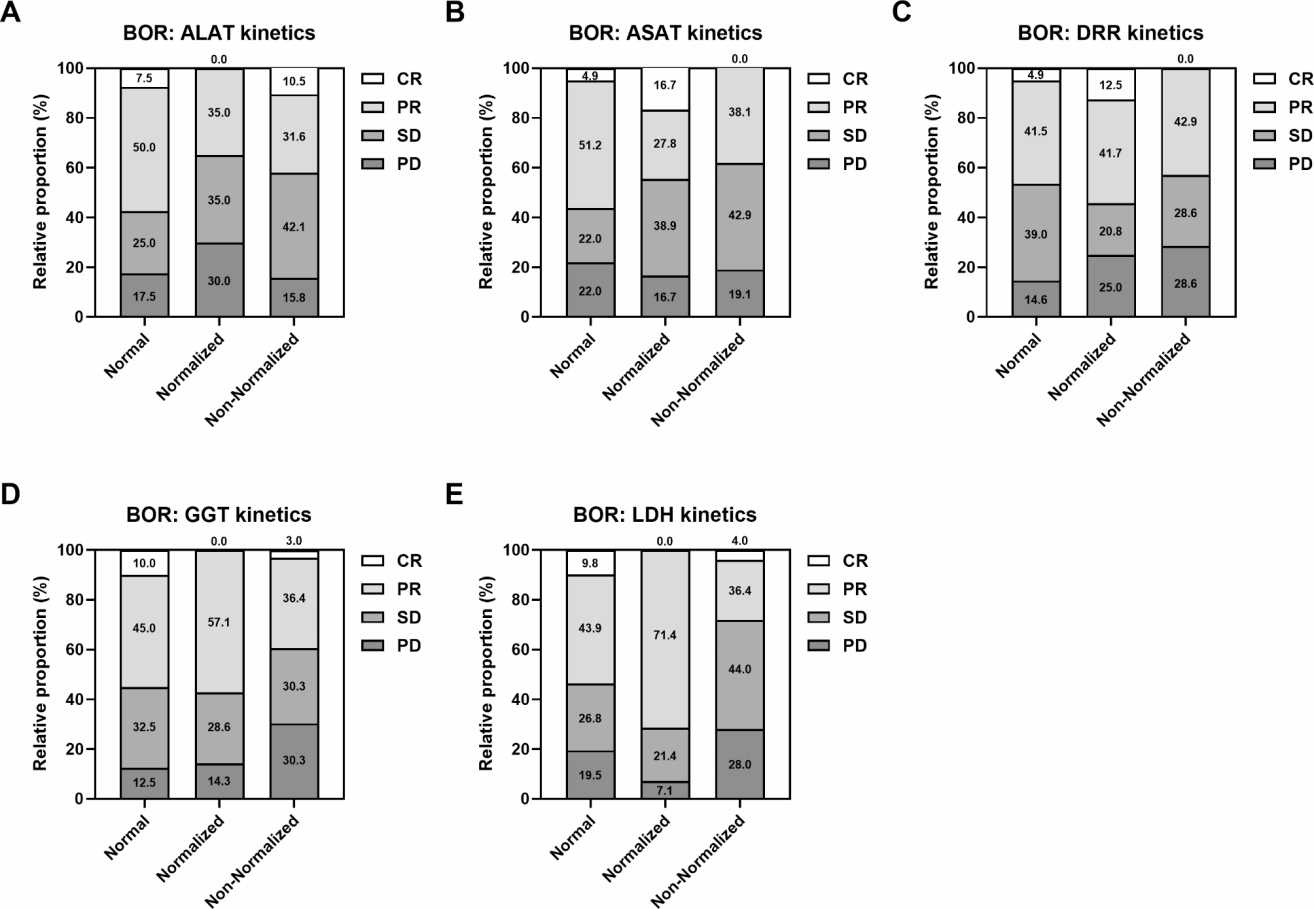
Association of baseline (**A**) ALAT, (**B**) ASAT, (**C**) DRR, (**D**) GGT, (**E**) LDH and (**F**) the grouped 4-Risk-Score with BOR of mRCC patients on CPI-based 1L therapy. Abbreviations: RF – risk factors.

**Fig. 5:**
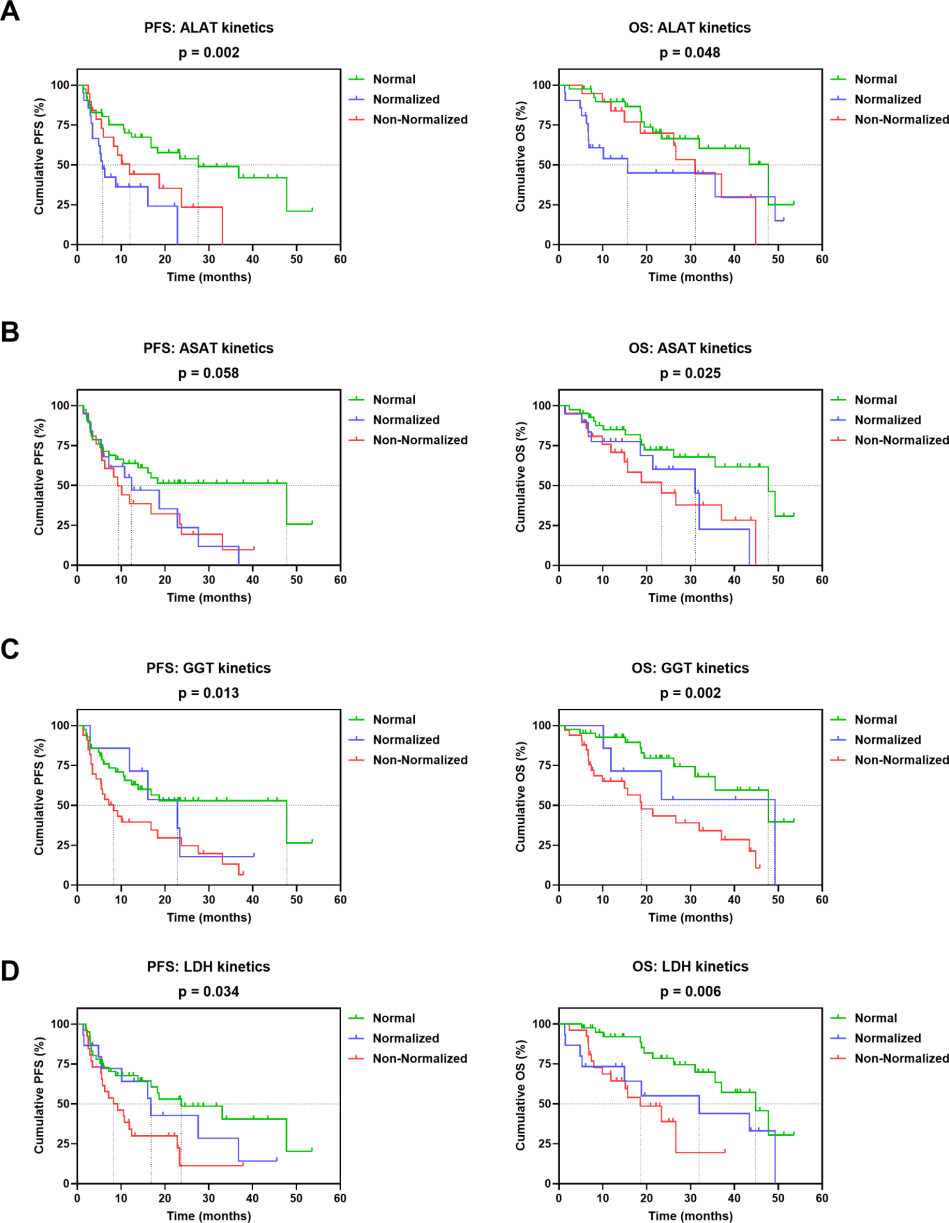
Association of early kinetics of (**A**) ALAT, (**B**) ASAT, (**C**) DRR, (**D**) GGT and (**E**) LDH with BOR of mRCC patients on CPI-based 1L therapy.

Next, we evaluated if baseline levels or early kinetics of the laboratory parameters and the grouped 4-Risk-Score were linked to the efficacy of CPI-based 1L therapy. Except for DRR, response to therapy (CR + PR) was more frequent in patients with low baseline values of the individual parameters or with a 4-Risk-Score of 0–1 or 2–3 (Fig. 4). Particularly, patients with low GGT and LDH had a higher rate of CR (~ 10%) than patients with high values did (~ 2.5%). In addition, patients with normal or normalized kinetics of the laboratory parameters mostly showed a higher frequency of response (Fig. 5). The highest proportion of CR (16.7%) were observed for normalized ASAT, while normalized LDH had the best overall response (71.4%).

## Discussion

In this real-world cohort of mRCC patients on CPI-based 1L therapy, we could show that elevated baseline levels of ALAT, ASAT, GGT and LDH were significantly associated with shorter PFS and OS. In contrast, the DRR as the ASAT/ALAT ratio did not provide an additional prognostic value. Baseline ALAT, ASAT, GGT and LDH were also identified as independent prognosticators in multivariate analysis. Particularly, GGT and LDH exhibited a higher prognostic value for OS than known clinico-pathological prognosticators, when their C-indices were compared. Furthermore, response to therapy was more frequent in patients with low baseline values of ALAT, ASAT, GGT and LDH.

In accordance, other studies have also shown that high baseline levels of GGT^23^and LDH^24–26^were independent prognosticators for worse survival in mRCC patients receiving ≥ 2L nivolumab. Consequently, these patients also exhibited lower response rates than patients with low GGT and LDH^23,24^. Similar results were observed in other tumor entities treated by CPI such as melanoma as well as lung and hepatocellular cancer^31–34^. In addition, elevated pre-treatment levels of GGT and LDH have also been identified as independent prognosticators for worse survival in mRCC patients undergoing TKI monotherapy^29,35^. In contrast, the study by Ekinci et al. could not observe an association between LDH and OS in a cohort of 75 mRCC patients on ≥ 2L nivolumab^36^. The authors stated that LDH was measured at the time of metastasis, but did not mention if this was at initial metastasis before 1L therapy or at progression on the treatment line immediately before nivolumab.

To the best of our knowledge, DRR has so far only been evaluated as a prognosticator for TKI- but not CPI-based therapy in mRCC patients. An increased DRR indicated worse survival in various studies^35,37,38^. Similar to our results, however, Janisch et al. observed no independent association between DRR and survival of TKI-treated mRCC patients^39^. Regarding the individual parameters ASAT and ALAT, only Kim et al. demonstrated that patients with high ASAT also had a poor prognosis, whereas ALAT was not identified as a prognosticator^38^. In accordance, high ASAT but not high ALAT levels were also associated with worse survival in hepatocellular cancer treated by CPI^34^. In the present study, however, ALAT had a slightly higher C-index for OS than ASAT and thus seems to be of prognostic importance for CPI-treated mRCC patients.

In addition to the individual markers, the combination of baseline ALAT, ASAT, GGT and LDH into the 4-Risk-Score was also identified as an independent prognosticator for PFS and OS. Patients with four risk factors displayed inferior survival and lower response rates as well as the highest risk for progression or death. Other studies have also shown that combining individual factors, e.g., LDH with lymphocyte-based ratios, can further stratify patients into prognostic groups^26,33,34^. In the present study, the 4-Risk-Score outperformed the prognostic value of known clinico-pathological prognosticators and added additional prognostic value over the individual laboratory parameters as indicated by a higher C-index for OS. Consequently, the 4-Risk-Score could be used to define prognostic groups to better stratify patients for CPI therapy. Importantly, all included parameters are cost-effective and readily available in the clinical routine.

Functionally, ALAT, ASAT, GGT and LDH are known to be involved in cancer metabolism and elevated baseline levels could indicate increased tumor burden and thus poor prognosis^12–14,16,17^. Due to their increased proliferation, cancer cells display metabolic reprogramming to ensure sufficient energy production and biosynthesis of key nutrients (e.g., amino acids and fatty acids). Compared to normal cells, malignant cells show an increased rate of aerobic glycolysis (Warburg effect), which leads to pyruvate production from glucose. LDH then catalyzes the conversion of pyruvate to lactate, while NADH is regenerated to NAD^+^, which can be reused in glycolysis^13^. Via lactate production, LDH also exerts immunosuppressive effects by influencing various immune cells^14^. Furthermore, ALAT and ASAT are also involved in glycolysis. As an important link between carbohydrate and amino acid metabolism, ALAT reversibly converts pyruvate into alanine, which serves as a major gluconeogenic precursor^15^. ASAT contributes to the relocation of NADH into mitochondria via the malate-aspartate-shuttle for subsequent NAD^+^regeneration^16,17^. GGT plays a fundamental role in the metabolism of glutathione, which is an important cellular antioxidant and mediates drug resistance during cytotoxic therapy^12^. GGT provides cysteine for the intracellular resynthesis of glutathione, thereby promoting the proliferation, migration and invasion of cancer cells including RCC cells^12,40^. Additionally, GGT might exert immunosuppressive functions^41^. Since some parameters positively correlated at baseline, ALAT, ASAT, GGT and LDH as well as their dynamic change might reflect cancer metabolism and tumor burden.

In accordance, the dynamic change of ALAT, ASAT, GGT and LDH in the early therapy phase was also associated with survival and CPI response. Normalization of elevated ASAT, GGT and LDH was linked to longer median survival and better response, whereas patients with normalized ALAT showed worse survival and lower response rates than the normal and non-normalized groups. Since ALAT is mainly produced in the liver and ASAT in various tissue types^42^, circulating ASAT levels might be more affected by metabolically altered cancer cells or their therapeutic response than ALAT levels. In turn, this could contribute to the divergent prognostic patterns of ALAT and ASAT kinetics. Furthermore, elevation of transaminase levels, particularly ALAT, during CPI therapy can indicate hepatotoxic irAEs^43^. In turn, the occurrence of irAEs often correlate with increased CPI efficacy^44^. Therefore, normalization of ALAT as indication of subsiding hepatotoxic irAEs could be associated with worse survival and response.

Overall, only normal ALAT, ASAT, GGT and LDH kinetics were identified as independent prognosticators for PFS and OS in multivariate Cox regression analyses. In a converse approach to ours, Ishiyama et al. showed that a ≥ 2-fold increase of GGT within 2 months compared to baseline was associated with poor prognosis in mRCC patients on ≥ 2L nivolumab^23^. Previously, we have demonstrated that normal and normalized kinetics of the inflammatory marker CRP were independent prognosticators for prolonged survival^10,11^. In summary, a normalization of elevated ASAT, GGT and LDH during the early treatment phase could indicate diminished tumor burden and metabolism, resulting in a better prognosis and CPI response. Such dynamic biomarkers in the early treatment phase could complement or even outperform static pre-treatment biomarkers (e.g., PD-L1 expression in primary tumor tissue), as they would reflect the complex mechanisms behind immune response more accurately^45^.

Several limitations to our study have to be discussed. First, the data were collected retrospectively from a small monocentric patient cohort. However, the present data stemmed from a real-world cohort including all available CPI-based 1L therapy options for mRCC, which can be considered a strength. Due to the study’s retrospective nature, the number of measurements for each laboratory parameter in the early therapy phase was highly variable (range 2–11). Particularly, patients with non-normalized kinetics could have been misclassified if the true nadir had not been determined due to lower numbers of blood sampling. In addition, other factors that could influence the blood levels of ALAT, ASAT, GGT and LDH, such as liver function, alcohol consumption and metabolic conditions, were not comprehensively surveyed. Furthermore, the cutoff values for baseline levels and subsequent classification of early kinetics were based on the respective median value of the laboratory parameter. The cutoff values for DRR, GGT and LDH used in other studies investigating their prognostic impact in CPI- or TKI-treated cancer patients ranged from 1.2 to 1.58, 0.7–1.2 µmol/s*l (40–71 U/l) and 3.3–4.4 µmol/s*l (196–264 U/l), respectively^23–26,31–39^, and thus were similar to our study (DRR: 1.18, GGT: 0.58 µmol/s*l = 35 U/l, LDH: 3.78 µmol/s*l = 227 U/l). However, optimal cutoff values should be defined in larger patient cohorts. Overall, the results on the prognostic value of ALAT, ASAT, GGT and LDH need to be confirmed prospectively in a larger multicentric study, which should also evaluate the influence of potential confounders (e.g., general liver dysfunction).

## Conclusion

Baseline levels and early kinetics of ALAT, ASAT, GGT and LDH were identified as important prognostic factors in mRCC patients treated with CPI-based 1L therapy. Compared to the individual parameters, combining the four parameters at baseline into a 4-Risk-Score resulted in an even better prognostic power. Three prognostic groups could be defined based on the 4-Risk-Score, which could help to better stratify patients for CPI therapy. Since all included parameters are cost-effective and readily available in the clinical routine, the 4-Risk-Score could be easily used for therapy monitoring of mRCC patients under CPI-based 1L therapy.

## Electronic supplementary material

Below is the link to the electronic supplementary material.

Supplementary Material 1

## Data Availability

The datasets generated during and/or analyzed during the current study are available from the corresponding author on reasonable request.
