# Supplementary material for: Prognostic potential of standard laboratory parameters in patients with metastatic renal cell cancer receiving first-line immunotherapy
Source: Sci Rep. 2024 Oct 25;14:25365. doi: 10.1038/s41598-024-76928-3 (PMC11511985; doi:10.1038/s41598-024-76928-3)
Supplement: Supplementary file 1 — Supplementary Material 1 [file 41598_2024_76928_MOESM1_ESM.pdf]

Scientific Reports (submitted in 2024) - Bjoern Thorben Buerk et al.

Prognostic potential of standard laboratory parameters in patients with metastatic renal cell cancer receiving first-line immunotherapy

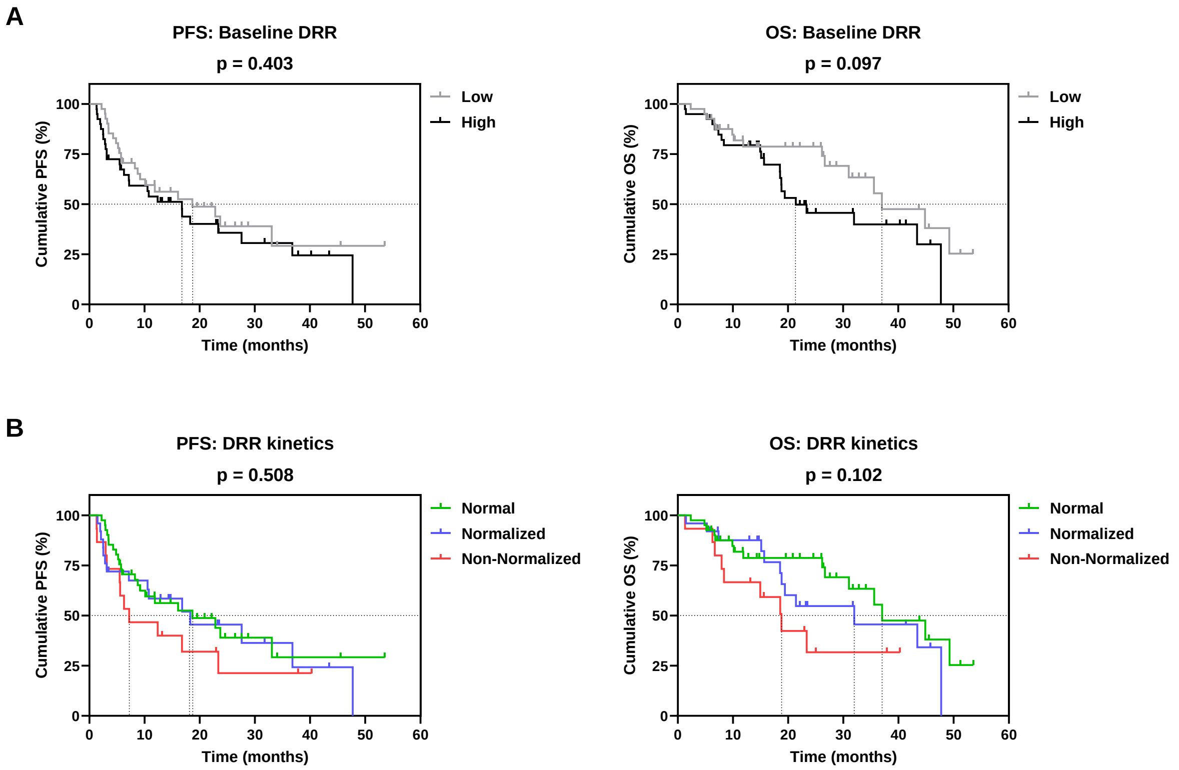

Fig. S1 Association of (A) baseline levels and (B) early kinetics of DDR with PFS and OS of mRCC patients on CPI-based 1L therapy. Vertical dashed lines indicate the respective median survival times of each category. P values were calculated by the log-rank test

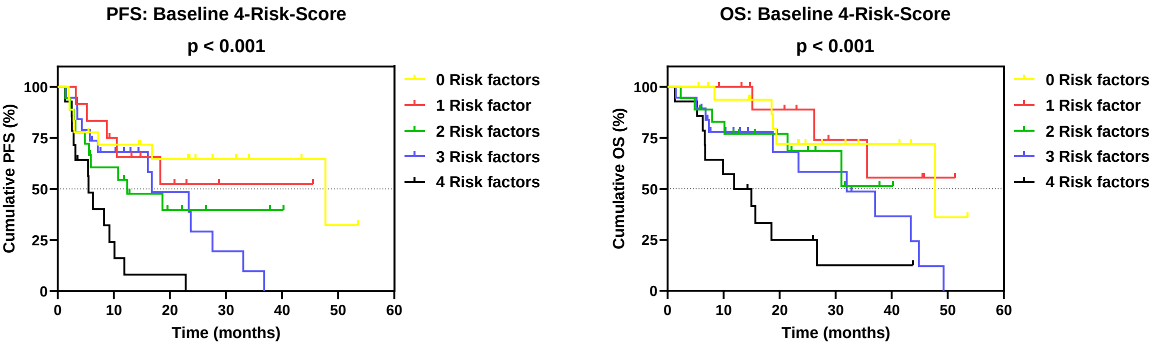

Baseline 4-Score	n (events)	1-year PFS	2-year PFS	Median PFS
0 Risk factors	18 (7)	71%	65%	47.7 mo.
1 Risk factor	12 (5)	66%	49%	n.r.
2 Risk factors	18 (10)	54%	37%	12.4 mo.
3 Risk factors	19 (13)	68%	30%	16.8 mo.
4 Risk factors	14 (13)	8%	0%	5.5 mo.

Baseline 4-Score	n (events)	1-year OS	2-year OS	Median OS
0 Risk factors	18 (5)	94%	70%	47.7 mo.
1 Risk factor	12 (3)	100%	90%	n.r.
2 Risk factors	18 (6)	76%	67%	n.r.
3 Risk factors	19 (11)	77%	58%	32.0 mo.
4 Risk factors	14 (11)	50%	26%	11.9 mo.

Fig. S2 Association of the baseline 4-Risk-Score with PFS and OS of mRCC patients on CPI-based 1L therapy. The table beneath each Kaplan-Meier curve includes the number of patients and events in each category as well as the respective median survival times, 1- and 2-year survival rates. P values were calculated by the log-rank test. Abbreviations: mo.: months; n.r.: not reached

Table S1 Pearson correlation coefficients (r) to indicate relations between two laboratory parameters at baseline

	**ALAT**	**ASAT**	**GGT**	**LDH**
**ALAT**	-			
**ASAT**	r = 0.652<br>**p < 0.001**<br>n = 81	-		
**GGT**	r = 0.453<br>**p < 0.001**<br>n = 81	r = 0.359<br>**p = 0.001**<br>n = 82	-	
**LDH**	r = 0.212<br>**p = 0.057**<br>n = 81	r = 0.440<br>**p < 0.001**<br>n = 82	r = 0.281<br>**p = 0.011**<br>n = 82	-

Significant p values (< 0.05) and statistical trends (p ≥ 0.05 and < 0.1) are displayed in bold

Table S2 Median baseline levels of ALAT, ASAT, DRR, GGT and LDH dependent on the presence of liver metastasis

**Parameter**	**No liver metastasis n = 71** [a]	**Liver metastasis n = 11**	**P value** [b]
**ALAT (µmol/s*l)**	0.31	0.34	0.452
**ASAT (µmol/s*l)**	0.36	0.40	0.320
**DRR**	1.17	1.22	0.381
**GGT (µmol/s*l)**	0.54	1.98	**0.006**
**LDH (µmol/s*l)**	3.70	4.38	0.128

[a] For ALAT and DRR n=70 due to missing baseline values for one patient

[b] Comparison between groups by the Mann-Whitney-U test

Significant p values (< 0.05) and statistical trends (p ≥ 0.05 and < 0.1) are displayed in bold

Table S3 Baseline and nadir levels as well as number of measurements according to early kinetics (normal, normalized, non-normalized) of ALAT, ASAT, DRR, GGT and LDH

Parameter	Category	Normal	Normalized	Non-Normalized	P value [c]
**ALAT** [a]					
Patients (n)		41 (50.6%)	21 (25.9%)	19 (23.5%)	
Baseline (µmol/s*l)	Median (range)	0.22 (0.09-0.32)	0.40 (0.33-1.23)	0.59 (0.34-1.48)	**<0.001**
Nadir (µmol/s*l) [b]	Median (range)	0.24 (0.08-2.10)	0.25 (0.15-0.31)	0.47 (0.33-1.36)	**<0.001**
Measurements (n)	Median (range)	5 (2-8)	6 (3-11)	7 (2-11)	**0.007**
**ASAT**					
Patients (n)		42 (51.2%)	19 (23.2%)	21 (25.6%)	
Baseline (µmol/s*l)	Median (range)	0.30 (0.14-0.37)	0.45 (0.38-0.95)	0.59 (0.42-2.72)	**<0.001**
Nadir (µmol/s*l) [b]	Median (range)	0.30 (0.08-0.77)	0.29 (0.21-0.37)	0.54 (0.39-1.17)	**<0.001**
Measurements (n)	Median (range)	5 (2-11)	6 (2-8)	6 (2-11)	0.333
**DRR** [a]					
Patients (n)		41 (50.6%)	25 (30.9%)	15 (18.5%)	
Baseline (µmol/s*l)	Median (range)	0.93 (0.44-1.18)	1.38 (1.20-2.93)	1.92 (1.33-3.69)	**<0.001**
Nadir (µmol/s*l) [b]	Median (range)	0.73 (0.19-1.57)	0.85 (0.38-1.18)	1.59 (1.28-6.53)	**<0.001**
Measurements (n)	Median (range)	6 (2-11)	6 (3-10)	5 (2-9)	**0.074**
**GGT**					
Patients (n)		42 (51.2%)	7 (8.5%)	33 (40.2%)	
Baseline (µmol/s*l)	Median (range)	0.42 (0.19-0.58)	0.85 (0.60-2.25)	1.88 (0.63-9.46)	**<0.001**
Nadir (µmol/s*l) [b]	Median (range)	0.34 (0.15-0.73)	0.44 (0.26-0.52)	1.25 (0.60-10.74)	**<0.001**
Measurements (n)	Median (range)	6 (2-11)	5 (4-7)	6 (3-11)	0.319
**LDH**					
Patients (n)		41 (50.0%)	15 (18.3%)	26 (31.7%)	
Baseline (µmol/s*l)	Median (range)	3.22 (1.97-3.76)	4.16 (3.79-5.65)	4.86 (3.79-16.49)	**<0.001**
Nadir (µmol/s*l) [b]	Median (range)	3.03 (1.44-5.63)	3.08 (1.94-3.72)	4.44 (3.79-10.75)	**<0.001**
Measurements (n)	Median (range)	5 (2-10)	6 (2-11)	5 (2-10)	0.106

[a] Baseline ALAT and thus DRR are missing for one patient

[b] Nadir within first three months after initiation of 1L therapy

[c] Comparison between groups of early kinetics of laboratory parameters by the Kruskal-Wallis test

Significant p values (< 0.05) and statistical trends ($p \geq 0.05$ and < 0.1) are displayed in bold

Table S4 Number of patients and events as well as median survival times, 1- and 2-year survival rates for PFS and OS depending on the respective baseline or early kinetics categories of ALAT, ASAT, GGT and LDH as well as the grouped 4-Risk-Score. Abbreviations: mo.: months

Parameter	Category	PFS				OS			
		n (events)	1-year PFS	2-year PFS	Median PFS	n (events)	1-year OS	2-year OS	Median OS
**Baseline ALAT**	Low	41 (20)	70%	54%	27.6 mo.	41 (14)	90%	65%	47.7 mo.
	High	40 (28)	39%	18%	8.8 mo.	40 (22)	68%	57%	26.7 mo.
**Baseline ASAT**	Low	42 (20)	63%	51%	47.7 mo.	42 (14)	85%	71%	47.7 mo.
	High	40 (29)	46%	22%	10.8 mo.	40 (22)	74%	52%	26.7 mo.
**Baseline DRR**	Low	41 (22)	56%	40%	18.7 mo.	41 (15)	79%	79%	37.0 mo.
	High	40 (26)	53%	36%	16.8 mo.	40 (21)	79%	45%	21.4 mo.
**Baseline GGT**	Low	42 (19)	66%	52%	47.7 mo.	42 (11)	93%	78%	47.7 mo.
	High	40 (30)	44%	22%	10.1 mo.	40 (25)	65%	45%	21.4 mo.
**Baseline LDH**	Low	41 (20)	67%	48%	23.7 mo.	41 (13)	92%	78%	44.9 mo.
	High	41 (29)	44%	26%	10.6 mo.	41 (23)	67%	46%	23.4 mo.
**Baseline 4-Risk-Score**	0-1 risk factors	30 (12)	69%	60%	47.7 mo.	30 (8)	96%	78%	47.7 mo.
	2-3 risk factors	37 (23)	61%	34%	16.8 mo.	37 (17)	77%	63%	32.0 mo.
	4 risk factors	14 (13)	8%	0%	5.5 mo.	14 (11)	50%	26%	11.9 mo.
**ALAT kinetics**	Normal	41 (20)	70%	54%	27.6 mo.	41 (14)	90%	65%	47.7 mo.
	Normalized	21 (15)	36%	9%	5.8 mo.	21 (12)	53%	45%	15.7 mo.
	Non-Normalized	19 (13)	44%	24%	11.9 mo.	19 (10)	84%	70%	31.0 mo.
**ASAT kinetics**	Normal	42 (20)	63%	51%	47.7 mo.	42 (14)	85%	71%	47.7 mo.
	Normalized	19 (13)	55%	23%	12.4 mo.	19 (9)	77%	59%	31.0 mo.
	Non-Normalized	21 (16)	40%	20%	9.2 mo.	21 (13)	71%	46%	23.4 mo.
**DRR kinetics**	Normal	41 (22)	56%	40%	18.7 mo.	41 (15)	79%	79%	37.0 mo.
	Normalized	25 (15)	58%	45%	18.3 mo.	25 (12)	88%	53%	32.0 mo.
	Non-Normalized	15 (11)	47%	23%	7.2 mo.	15 (9)	67%	32%	18.8 mo.
**GGT kinetics**	Normal	42 (19)	66%	52%	47.7 mo.	42 (11)	93%	78%	47.7 mo.
	Normalized	7 (5)	71%	19%	22.8 mo.	7 (4)	71%	54%	49.3 mo.
	Non-Normalized	33 (25)	38%	23%	8.3 mo.	33 (21)	64%	43%	18.8 mo.
**LDH kinetics**	Normal	41 (20)	67%	48%	23.7 mo.	41 (13)	92%	78%	44.9 mo.
	Normalized	15 (9)	65%	46%	16.8 mo.	15 (9)	73%	55%	32.0 mo.
	Non-Normalized	26 (20)	34%	16%	8.3 mo.	26 (14)	64%	41%	18.5 mo.

Table S5 Univariate Cox regression analyses for PFS and OS dependent on baseline levels and early kinetics of ALAT, ASAT, DRR, GGT and LDH as well as the grouped 4-Risk-Score

Parameter	Category	PFS univariate			OS univariate		
		HR (95% CI)	P value	C-index	HR (95% CI)	P value	C-index
**Baseline ALAT**	Low<br>High	Reference<br>2.61 (1.43-4.92)	**0.002**	0.598	Reference<br>2.07 (1.07-4.16)	**0.034**	0.610
**Baseline ASAT**	Low<br>High	Reference<br>2.00 (1.12-3.64)	**0.020**	0.550	Reference<br>2.58 (1.29-5.44)	**0.009**	0.593
**Baseline DRR**	Low<br>High	Reference<br>1.28 (0.72-2.27)	0.403	0.540	Reference<br>1.77 (0.90-3.58)	0.101	0.563
**Baseline GGT**	Low<br>High	Reference<br>2.18 (1.23-3.99)	**0.009**	0.580	Reference<br>2.99 (1.50-6.36)	**0.003**	0.645
**Baseline LDH**	Low<br>High	Reference<br>1.91 (1.08-3.48)	**0.029**	0.569	Reference<br>2.75 (1.40-5.62)	**0.004**	0.653
**Baseline 4-Risk-Score**	0-1 risk factors<br>2-3 risk factors<br>4 risk factors	Reference<br>2.10 (1.04-4.49)<br>5.97 (2.58-14.1)	 **0.044**<br>**<0.001**	0.629	Reference<br>2.56 (1.13-6.32)<br>6.84 (2.67-18.4)	 **0.031**<br>**<0.001**	0.693
**ALAT kinetics**	Non-Normalized<br>Normal<br>Normalized	Reference<br>0.47 (0.23-0.99)<br>1.59 (0.75-3.42)	 **0.041**<br>0.231	0.618	Reference<br>0.60 (0.27-1.39)<br>1.56 (0.65-3.80)	 0.214<br>0.313	0.647
**ASAT kinetics**	Non-Normalized<br>Normal<br>Normalized	Reference<br>0.48 (0.25-0.95)<br>0.91 (0.43-1.90)	 **0.031**<br>0.807	0.556	Reference<br>0.37 (0.17-0.83)<br>0.92 (0.38-2.14)	 **0.014**<br>0.842	0.599
**DRR kinetics**	Non-Normalized<br>Normal<br>Normalized	Reference<br>0.65 (0.32-1.40)<br>0.74 (0.34-1.66)	 0.247<br>0.448	0.552	Reference<br>0.40 (0.17-0.98)<br>0.59 (0.25-1.46)	 **0.037**<br>0.239	0.587
**GGT kinetics**	Non-Normalized<br>Normal<br>Normalized	Reference<br>0.41 (0.22-0.75)<br>0.60 (0.20-1.45)	 **0.004**<br>0.300	0.596	Reference<br>0.29 (0.13-0.59)<br>0.51 (0.14-1.41)	 **0.001**<br>0.242	0.663
**LDH kinetics**	Non-Normalized<br>Normal<br>Normalized	Reference<br>0.44 (0.23-0.83)<br>0.62 (0.27-1.34)	 **0.011**<br>0.244	0.586	Reference<br>0.30 (0.13-0.66)<br>0.65 (0.25-1.58)	 **0.003**<br>0.353	0.653

Significant p values (<0.05) and statistical trends ($p \geq 0.05$ & <0.1) are displayed in bold. HR: hazard ratio, CI: confidence interval
